# Gene Mapping and Gene-Set Analysis for Milk Fever Incidence in Holstein Dairy Cattle

**DOI:** 10.3389/fgene.2018.00465

**Published:** 2018-10-10

**Authors:** Hendyel A. Pacheco, Simone da Silva, Anil Sigdel, Chun Kuen Mak, Klibs N. Galvão, Rodrigo A. Texeira, Laila T. Dias, Francisco Peñagaricano

**Affiliations:** ^1^Department of Animal Sciences, University of Florida, Gainesville, FL, United States; ^2^Departamento de Zootecnia, Universidade Federal do Paranaì, Curitiba, Brazil; ^3^Department of Large Animal Clinical Sciences, University of Florida, Gainesville, FL, United States; ^4^University of Florida Genetics Institute, University of Florida, Gainesville, FL, United States

**Keywords:** calcium metabolism, enrichment analysis, periparturient hypocalcemia, vitamin D metabolism, whole-genome scan

## Abstract

Milk fever is an important metabolic disorder that affects dairy cows around parturition. It is associated with a breakdown in the mechanisms of calcium homeostasis, resulting in very low blood calcium levels (hypocalcemia). The main objective of this study was to dissect the genetic basis underlying milk fever incidence in Holstein cattle. Data consisted of 31.6 k producer-recorded lactation incidence records from 15.3 k cows. The analysis included a whole-genome scan and a subsequent gene-set analysis in order to reveal individual genes, genetic mechanisms and biological pathways implicated in the incidence of periparturient hypocalcemia. The association analysis identified at least eight different genomic regions that explain considerable amounts of additive genetic variance for milk fever incidence. Notably, some of these regions harbor genes, such as *CYP27A1, CYP2J2, GC, SNAI2*, and *PIM1*, that are directly involved in vitamin D metabolic pathway. Moreover, the gene-set analysis revealed several functional terms, such as calcium ion binding, calcium ion transportation, T cell differentiation, B cell activation, protein phosphorylation, apoptosis, and protein kinase activity, among others, that could be implicated in the development of periparturient hypocalcemia. Overall, this comprehensive study contributes to a better understanding of the genetic control of this complex disease. In addition, these findings may contribute to the development of novel breeding strategies for reducing the incidence of milk fever in dairy cattle.

## Introduction

Periparturient hypocalcemia or periparturient paresis, commonly known as milk fever, is a metabolic disorder that affects dairy cows around parturition. The onset of lactation demands a huge amount of calcium for colostrum and milk synthesis, and some cows are unable to adapt to this demand and succumb to clinical hypocalcemia (Horst et al., 1997, 2005). Indeed, milk fever arises when the homeostatic mechanisms fail to maintain normal blood calcium concentrations in early lactation. Clinical symptoms include partial to complete paralysis (downer cows) and reduced feed intake. The occurrence of milk fever predisposes the cow to other metabolic and infectiousdisorders, such as retained placenta, uterine prolapse, endometritis, displaced abomasum, ketosis, and mastitis (Mulligan et al., 2006). Economic losses are substantial and include losses due to on-farm death, premature culling, reduced milk production, and increased veterinary and treatment costs (Liang et al., 2017).

There is growing evidence that milk fever is influenced by genetic factors. Indeed, the incidence of milk fever is a low to moderate heritable trait in dairy cattle, with heritability estimates ranged from 0.01 to 0.35 depending on the breed and the methodology (Pryce et al., 2016). The magnitude of these heritability estimates suggests that genetic selection may be effective. In addition, it is well-documented that breed has a significant effect on the risk of milk fever, particularly Swedish Red and White and Jersey cows are known to be more susceptible to succumb to clinical hypocalcemia than Holstein cows (Kusumanti et al., 1993; Lean et al., 2006). Furthermore, high-throughput technologies, including massively parallel metabolite and protein quantification, have revealed numerous differences between healthy and milk fever cows (Xia et al., 2012; Shu et al., 2014; Sun et al., 2014). Overall, current evidence suggests that genetic factors may explain part of the differences in susceptibility to periparturient hypocalcemia among dairy cows, and hence, this metabolic disorder could be improved by genetic means.

There is very limited information on genes associated with risk of milk fever in dairy cattle. Using a multiple-marker mapping approach, Elo et al. (1999) identified a genomic region on chromosome 23 significantly associated with veterinary treatment, including milk fever, in Finnish Ayrshire dairy cattle. Furthermore, Sasaki et al. (2014), using a microarray-based gene expression study of peripheral blood mononuclear cells, suggested genes *PKIB, DDIT4, PER1*, and *NUAK1* as potential biomarkers for milk fever predisposition.

The identification of individual genes and gene networks affecting milk fever incidence could have multiple benefits, including better understanding of the biology underlying this complex metabolic disorder, promote the development of new drugs and therapies, and contribute to the design of novel strategies for improving milk fever via selective breeding. As such, the objective of the present study was to perform a whole-genome scan and a subsequent gene-set enrichment analysis combining producer-recorded health event records and high-density single nucleotide polymorphism (SNP) data in order to identify gene and gene-sets underlying milk fever incidence in Holstein cattle.

## Materials and Methods

### Phenotypic and Genotypic Data

Data consisted of 31,618 producer-recorded lactation incidence records of milk fever from 15,290 Holstein cows that calved between January 2010 and December 2017 in two dairy herds in the State of Florida, United States. Milk fever data was recorded as binary, i.e., Y = 1 if the cow had clinical symptoms of severe hypocalcemia (downer cow) during the first 48 h after parturition, and Y = 0 otherwise. Three-point four percent of the cows had at least one case of milk fever. The incidence was very low for cows in first (0.07%) and second lactation (0.30%), but increased progressively with parity, reaching 11.2% for cows in 5th or later lactation.

Genotype data for 60,671 single nucleotide polymorphism (SNP) markers were available for 7,052 cows with health records and also 1,498 sires in the pedigree. Those SNP markers that mapped to the sex chromosomes, or were monomorphic, or had minor allele frequency less than 1% were removed from the SNP dataset. After data editing, a total of 58,009 SNP markers were retained for subsequent genomic analyses.

### Statistical Models

The incidence of milk fever was analyzed using a probit model. The probit model, also known as threshold model (Gianola, 1982) describes the observable response variable (Y, either 0 or 1) using an underlying linear model,

z=η+ε

where η is a vector of linear predictors and _ξ_ is a vector of independent and identically distributed standard normal random variables. In this context, the observable outcome is Y = 1 if the underlying z variable (liability) is greater than zero, i.e., Y = {1 if z > 0; 0 otherwise}. Therefore, the conditional probability of observing a milk fever event is P(Y = 1|η) = ϕ(η) where ϕ(⋅) is the standard normal cumulative distribution function. The likelihood function then becomes:

$$p(Y|\text{η})=\prod \phi {\left(\text{η}\right)}^{Y}\cdot {\left[1-\phi (\text{η})\right]}^{1-Y}$$

where Y and η are vector of response variables and the linear predictors, respectively.

For milk fever incidence, the linear predictor η has the following form:

$$\text{η}=X\text{β}+Z_{1}hys+Z_{2}u+Wpe$$

where β is a vector of fixed effects in the model, hys is a vector of random herd-year-season effects (56 levels), u is a vector of random additive genetic effects (34,175 levels), and pe is a vector of random permanent environmental effect (15,290 levels). The vector β includes the intercept and the lactation number as class variable with 5 levels (1, 2, 3, 4, and 5+). The matrices X, Z_1_, Z_2_, and W are the incidence matrices relating phenotypic records to fixed, herd-year-season, animal and permanent environmental effects, respectively. Random effects were assumed to follow a multivariate normal distribution,

$$\left(\begin{array}{c} hys\\ u\\ pe \end{array}|{\text{σ}}_{hys}^{2},{\text{σ}}_{u}^{2},{\text{σ}}_{pe}^{2}\right)\sim N\left[0,\left(\begin{array}{ccc} I{\text{σ}}_{hys}^{2} & 0 & 0\\ 0 & H{\text{σ}}_{u}^{2} & 0\\ 0 & 0 & I{\text{σ}}_{pe}^{2} \end{array}\right)\right]$$

where ${\text{σ}}_{hys}^{2},{\text{σ}}_{u}^{2}{\text{ and σ}}_{pe}^{2}$ are the herd-year-season, additive genetic, and permanent environmental variances, respectively. Here, the classical pedigree relationship matrix A is replaced by H which combines pedigree and genotypic information (Aguilar et al., 2010). This method is known as single-step genomic best linear unbiased prediction (ssGBLUP). The combined pedigree-genomic relationship matrix H^-1^ was calculated as follows,

$$H^{-1}=A^{-1}+\left[\begin{array}{cc} 0 & 0\\ 0 & G^{-1}-A_{22}^{-1} \end{array}\right]$$

where G^-1^ is the inverse of the genomic relationship matrix and $A_{22}^{-1}$ is the inverse of the pedigree-based relationship matrix for genotyped animals. In this case, **G** has a dimension 8,550 × 8,550 and was created considering 7,052 cows with both genotypes and milk fever records plus 1,498 genotyped sires in the pedigree. The pedigree relationship **A** matrix (34,175 × 34,175) was created based on a five-generation pedigree file obtained from the Council of Dairy Cattle Breeding.

### Implementation

The threshold model was implemented in a Bayesian framework using THRGIBBS1F90 (Tsuruta and Misztal, 2006). Given that the fully conditional posterior distribution of this model does not have a close form, the Gibbs sampler was used to study features of the posterior distribution. Inferences were based on 800,000 samples obtained after discarding the first 200,000 samples as burn in. A thinning interval of 100 was used to compute statistics of the posterior distribution. Convergence diagnostics of Markov chain Monte Carlo sampling output were carried out by visual inspection of trace plots of some parameters, such as variance components.

### Gene Mapping

The single-step GBLUP is one of a group of methods that were initially developed for performing genomic prediction and later were extended for performing gene mapping. Candidate genomic regions associated with milk fever were identified based on the amount of genetic variance explained by 2.0 Mb window of adjacent SNPs. Given the genomic estimated breeding values (GEBVs), the SNP effects can be estimated as $\hat{m}=DM'{\left[MDM'\right]}^{-1}\hat{a_{g}}$, where $\hat{m}$ is the vector of SNP marker effects, D is a diagonal matrix of weights of SNPs, **M** is a matrix relating genotypes of each locus, and $\hat{a_{g}}$ is the vector of GEBVs (Wang et al., 2012). In this study, the SNP were equally weighted. The percentage of genetic variance explained by a given 2.0 Mb region was then calculated as,

var(ui)σu2×100=var(∑j=1NMjmj)σu2×100

where u_i_ is the genetic value of the i^th^ genomic region under consideration, N is the total number of adjacent SNPs within the 2.0 Mb genomic region, and m_j_ is the marker effect of the j^th^ SNP within the i^th^ region. These calculations were performed using the program POSTGSF90 (Aguilar et al., 2014).

### Gene-Set Enrichment Analysis

Overrepresentation or gene-set enrichment analysis has proven to be a great complement of gene mapping (e.g., Gambra et al., 2013; Abdalla et al., 2016). As described in detail by Han and Peñagaricano (2016), a gene-set analysis consists basically in three steps: (i) the assignment of SNP markers to annotated genes, (ii) the assignment of genes to gene-sets, and finally (iii) the association analysis between each gene-set and the phenotype of interest.

Using the UMD3.1 bovine genome sequence assembly (Zimin et al., 2009), a given SNP marker was assigned to a particular annotated gene if the SNP was located within the genomic sequence of the gene or at most 10 kb upstream the gene. An arbitrary threshold of 2% of the SNP effects distribution (in absolute value) was used to define the set of relevant SNP markers; in this context, putative genes affecting milk fever incidence were defined as those genes that contained at least one relevant SNP. The Gene Ontology (GO) database (Ashburner et al., 2000) was used to define functional gene-sets, i.e., group of genes that share some particular properties, typically their involvement in the same biological process or molecular function. Finally, the association between a particular gene-set and milk fever incidence was evaluated using a Fisher’s exact test (Peñagaricano et al., 2013).

## Results and Discussion

Milk fever is the most important macromineral disorder affecting dairy cows around parturition due to low level of calcium in the blood. Milk fever is also considered a gateway disease because the occurrence of periparturient hypocalcemia is related to increased incidence rates of other transition disorders, such as retained placenta, metritis and mastitis. Despite its economic importance, little is known about genetic factors affecting the risk of milk fever. This study was specifically conducted to unravel individual genes, functional gene-sets and biological pathways underlying susceptibility to milk fever in Holstein dairy cows.

### Whole-Genome Scan

**FIGURE 1:**
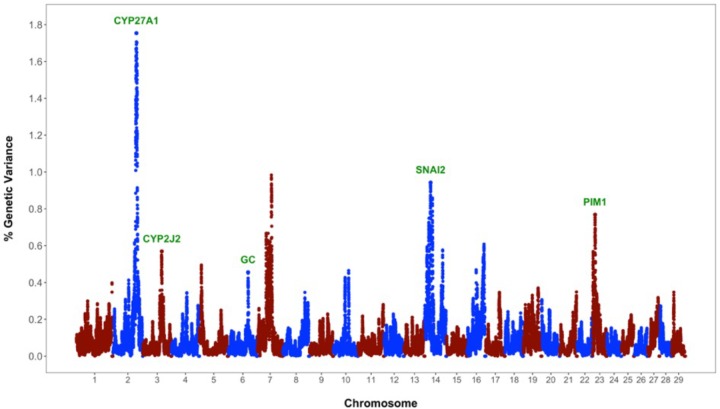
Whole-genome scan for milk fever incidence in Holstein dairy cattle: Percentage of additive genetic variance explained by 2.0 Mb single nucleotide polymorphism (SNP)-windows across the genome. Genes directly implicated in vitamin D metabolism and signaling are highlighted in green.

The identification of genomic regions and putative genes affecting periparturient hypocalcemia ($\hat{h^{2}}=0.128$) was performed using the single-step genomic BLUP. This method combines all the available phenotypic, pedigree and genotypic information, and fits all the SNP simultaneously. Candidate regions and genes were identified based on the amount of genetic variance explained by 2.0 Mb SNP-windows across the entire bovine genome (**Figure 1**). A total of eight different genomic regions, located on chromosomes BTA2, BTA3, BTA5, BTA6, BTA7, BTA14, BTA16, and BTA23, explain more than 0.45% of the additive genetic variance for milk fever incidence (**Table 1**). Interestingly, five of these regions harbor genes, namely *CYP27A1* (BTA2, 107–109 Mb), *CYP2J2* (BTA3, 86–88 Mb), *GC* (BTA6, 87–89 Mb), *SNAI2* (BTA14, 19.5–21.5 Mb), and *PIM1* (BTA23, 10.5–12.5 Mb), that play critical roles in vitamin D metabolism and signaling (**Figure 1**). In addition, the candidate region identified on BTA7 (62.5–64.5 Mb) harbors two candidate genes, *CAMK2A* and *ANXA6*, that are implicated in calcium ion transport.

**Table 1:** Genomic regions (2-Mb single nucleotide polymorphism (SNP)-windows) associated with milk fever incidence.

Chromosome	Position (Mb)	Genetic variance (%)	Candidate genes
BTA2	107–109	1.75	*CYP27A1*
BTA3	86–88	0.57	*CYP2J2*
BTA5	6.5–8.5	0.50	*-*
BTA6	87–89	0.46	*GC*
BTA7	62.5–64.5	0.98	*CAMK2A, ANXA6*
BTA14	19.5–21.5	0.94	*SNAI2*
BTA16	77–79	0.61	*-*
BTA23	10.5–12.5	0.77	*PIM1*

**FIGURE 2:**
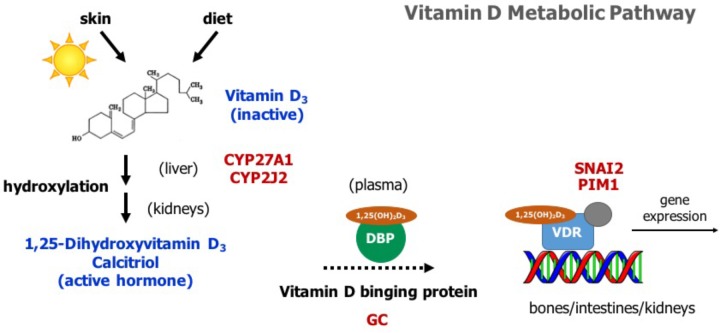
Overview of the vitamin D metabolic pathway. Vitamin D_3_ is obtained from the diet or by photoconversion in the skin. It is converted to its active form, 1,25-dihydoxyvitamin D_3_ or calcitriol, by two consecutive hydroxylation steps, first in the liver and then in the kidneys. Calcitriol travels to its target organs (bones, intestines, kidneys) bound to vitamin D binding protein (DBP). It mediates its biological effects by activating the vitamin D receptor (VDR), which in turn modulates the expression of several genes involved in calcium homeostasis. Genes identified in the whole-genome scan are highlighted in red.

Vitamin D has a significant role in calcium homeostasis. Basically, it controls blood calcium levels by promoting calcium absorption in the intestines, calcium reabsorption in the kidneys, and bone resorption (Horst et al., 2005). **Figure 2** provides an overview of the vitamin D metabolic pathway. Vitamin D_3_, also known as cholecalciferol, is the predominant form of vitamin D in cattle. Cholecalciferol is obtained from the diet or is synthesized in the skin from 7-dehydrocholesterol by ultraviolet irradiation (Horst et al., 1994). Vitamin D_3_ is inactive and the conversion to its active form, 1,25-dihydoxyvitamin D_3_, commonly known as calcitriol, involved two consecutive hydroxylation steps effected by members of the cytochrome P450 superfamily of enzymes (**Figure 2**). The first hydroxylation occurs in the liver and is mediated by several vitamin D-25-hydroxylases, including the mitochondrial CYP27A1 and the microsomal forms CYP2R1, CYP3A4, and CYP2J2 (Schuster, 2011). Notably, our whole-genome scan detected *CYP27A1* and *CYP2J2* as two putative genes affecting milk fever incidence (**Figure 1**). Although CYP2R1 is considered the key enzyme responsible for the conversion of vitamin D_3_ to 25-hydroxyvitamin D_3_ in humans and rodents, our results suggest that CYP27A1 and CYP2J2 may play also an important role in cattle. Indeed, gene *CYP2J2* has been associated with serum levels of 25-hydroxyvitamin D_3_ in beef cattle (Casas et al., 2013).

Vitamin D_3_ and all its metabolites, including the major circulating metabolite 25-hydroxyvitamin D_3_, and the active form 1,25-dihydoxyvitamin D_3_, are transported in the plasma by vitamin D binding protein (DBP) (**Figure 2**). The DBP, originally known as Gc-globulin, is a member of the albumin protein family (Haddad, 1995). It is predominantly synthesized in the liver and circulates in the plasma at high concentrations, and thus, under normal physiological conditions, nearly all circulating vitamin D_3_ metabolites are protein bound (White and Cooke, 2000). Remarkably, protein DBP is encoded by the gene *GC*, and gene *GC* was found as a candidate gene underlying milk fever in our whole-genome association analysis (**Figure 1**). Genetic variants in *GC* can either change the structure or the abundance of DBP, which in turn can greatly impact the function of the entire vitamin D system. In fact, *GC* is highly polymorphic in humans, with three common alleles and more than 120 rare variants, and several studies have linked some of these polymorphisms and susceptibility or resistance to several diseases (Speeckaert et al., 2006). Our findings provide evidence that polymorphisms in *GC* gene can also impact health performance in dairy cattle.

Calcitriol (1,25-dihydoxyvitamin D_3_) mediates its biological effects in its target organs, such as intestines, kidneys, and bones, by activating the vitamin D receptor (VDR) (**Figure 2**). The VDR is a member of the nuclear hormone receptor superfamily and operates as a ligand-inducible transcription factor (Haussler et al., 2013). The activated 1,25-dihydoxyvitamin D_3_-VDR complex interacts with the retinoid X receptor (RXR) to form a heterodimer that binds to specific DNA sequence motifs in the regulatory region of vitamin D target genes. This activated heterodimer together with several coregulatory protein complexes modulate the transcription of genes that mediate vitamin D functions, including control of calcium homeostasis (Pike et al., 2007). Interestingly, two candidate genes identified in the association analysis, namely *SNAI2* and *PIM1*, are directly implicated in VDR-mediated regulation of gene expression (**Figure 1**). On the one hand, SNAI2 is a zinc finger transcription factor that represses VDR gene expression, and thereby blocks calcitriol function (Larriba et al., 2009). On the other hand, PIM1, a serine/threonine kinase of the calcium/calmodulin-regulated kinase family, interacts directly with VDR and coactivates the expression of vitamin D target genes (Maier et al., 2012).

Some of the current strategies to reduce periparturient hypocalcemia in dairy cattle include feeding acidogenic diets supplemented with vitamin D metabolites (Martinez et al., 2018a,b). Our findings suggest that targeting vitamin D metabolic pathway via marker-assisted selection could be an alternative strategy to improve postpartum health. Future functional studies, e.g., resequencing and fine mapping, are needed to decipher the specific roles of the vitamin D system. Although current breeding programs include milk fever (Cole and VanRaden, 2018), the use of major genes and causative mutations could accelerate genetic gains.

### Gene-Set Analysis

The whole-genome scan successfully detected some major regions associated with milk fever incidence, including five genes directly implicated in vitamin D activation, transportation, and signaling. However, it should be recognized that these genomic regions explain only part of the genetic variance, and hence, a complementary approach is needed to fully reveal the genetic architecture underlying this complex phenotype. As such, a gene-set enrichment analysis using GO database was used in order to obtain additional insights regarding biological pathways and genetic mechanisms affecting this metabolic disorder.

**FIGURE 3:**
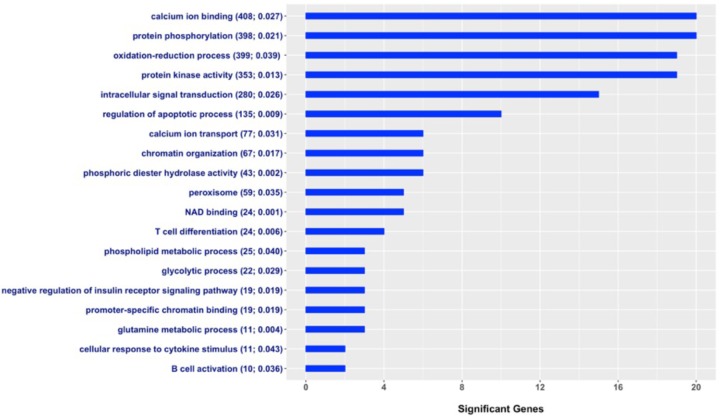
Gene Ontology (GO) gene-set terms significantly enriched with genes associated with milk fever incidence in Holstein dairy cattle. The graph shows the number of genes associated with periparturient hypocalcemia per each significant gene-set; total number of genes in the GO term and *P*-value from the Fisher’s exact test are displayed in parenthesis.

A total of 22,246 of the 58,009 evaluated SNPs were located within or at most 10 kb upstream of 12,126 annotated genes in the UMD 3.1 bovine genome assembly. A subset of 365 of these 12,126 genes were defined as associated with milk fever incidence (**Supplementary Table S1**). The enrichment analysis, i.e., search for gene-sets that show an overrepresentation of candidate genes, was performed using Fisher’s exact test, a test of proportions based on the cumulative hypergeometric distribution. **Figure 3** shows a set of GO terms that were significantly enriched with genes associated with milk fever incidence. These functional terms are related to calcium homeostasis, immune response, gene expression regulation, energy metabolism, protein kinase activity, and protein phosphorylation, among others. The entire list of significant gene-sets can be found in **Supplementary Table S2**.

Two gene-sets closely related to calcium metabolism, *calcium ion binding* and *calcium ion transport*, showed significant enrichment with genes associated with milk fever incidence. The onset of lactation increases irreversible losses of calcium in colostrum and milk, and when cows are unable to adapt to this demand, they develop milk fever (Goff, 2008). Unsurprisingly, our findings show that susceptibility to periparturient hypocalcemia in dairy cows is determined in part by genes directly related to calcium homeostasis, such as calcium sensors and calcium transporters. Furthermore, many significant GO terms were associated with immune response, such as *T cell differentiation, B cell activation*, and *cellular response to cytokine stimulus*. It has been shown that hypocalcemia is associated with impaired immune response in dairy cows (Kimura et al., 2006; Martinez et al., 2014). Our results suggest that alterations in the immune system could be part of the causes of milk fever. Interestingly, a recent study has shown that cows affected by milk fever showed alterations in the innate immunity several weeks prior to the appearance of clinical signs of hypocalcemia (Zhang et al., 2018). Moreover, terms related to protein phosphorylation, regulation of apoptotic process, gene expression regulation, and intracellular signal transduction were also enriched with candidate genes for milk fever incidence. Of particular interest, Sasaki et al. (2014) investigated gene expression of peripheral blood mononuclear cells in milk fever vs. healthy cows. Notably, upregulated genes were involved in regulation of protein amino acid phosphorylation, intracellular signaling cascade, apoptosis, and cell death, while downregulated genes were implicated in cation-binding, transcription regulation, and DNA-binding (Sasaki et al., 2014; Dervishi and Ametaj, 2017). Therefore, our results provide further evidence that processes such as protein phosphorylation and regulation of apoptosis, among others, could be implicated in the development of periparturient hypocalcemia in dairy cows.

Although much is known about preventing and treating milk fever in dairy cows, the real cause of failure in the calcium homeostatic mechanisms remains unclear. Our gene-set enrichment analysis suggests that there are many genetic mechanisms and biological pathways underlying this complex metabolic disorder in dairy cattle. More in general, these findings provide evidence that gene-set analyses can greatly complement genomic scans in understanding biological processes and molecular mechanisms affecting complex phenotypes.

## Conclusion

We performed an integrative genomic analysis in order to reveal the genetic basis of periparturient hypocalcemia, commonly known as milk fever, in Holstein dairy cattle. Eight genomic regions, located on chromosomes BTA2, BTA3, BTA5, BTA6, BTA7, BTA14, BTA16, and BTA23, explained considerable amounts of additive genetic variance. Interestingly, five of these regions harbor genes, namely *CYP27A1, CYP2J2, GC, SNAI2*, and *PIM1*, that are directly implicated in vitamin D activation, transportation, and signaling. Vitamin D controls calcium homeostasis, and hence, it could play a major role in preventing milk fever in dairy cattle. Moreover, the enrichment analysis revealed functional gene-sets, such as calcium ion binding, calcium ion transportation, T cell differentiation, B cell activation, protein phosphorylation, apoptosis, among others, that could be implicated in the development of periparturient hypocalcemia. Overall, this comprehensive study unraveled putative genes and biological mechanisms responsible for the genetic variation in milk fever incidence. Our findings can provide opportunities for improving periparturient hypocalcemia in dairy cattle via marker-assisted selection.

## Data Accessibility

The phenotypic and genotypic data analyzed in this study were obtained from North Florida Holsteins (Bell, FL, United States), University of Florida Dairy Research Unit (Alachua, FL, United States), and Council of Dairy Cattle Breeding (Bowie, MD, United States). These datasets were used under agreement, and hence, are not publicly available. However, data are available upon request to FP and with permission of North Florida Holsteins, University of Florida Dairy Research Unit, and Cooperative Dairy DNA Repository.

## Author Contributions

FP and KG conceived and designed the study. CM and AS collected the data. HP, SdS, AS, and FP conducted the statistical analyses. KG, RT, and LD supervised the study and contributed to the interpretation of the results. HP and FP wrote the first draft of the manuscript. All the authors read and approved the manuscript before submission.

## Conflict of Interest Statement

The authors declare that the research was conducted in the absence of any commercial or financial relationships that could be construed as a potential conflict of interest.

## Supplementary Material

TABLE S1 List of single nucleotide polymorphism (SNPs) in the 2% of the SNP effects distribution.

TABLE S2 List of significant gene-set terms.
